# Peer-led, faith-based co-designed intervention to improve Muslim women’s cancer screening: results from the IMCAN study

**DOI:** 10.3389/fpubh.2026.1918791

**Published:** 2026-10-09

**Authors:** Floor Christie-De Jong, Rawand Jarrar, Aasim I. Padela, P. Theo Pepler, Marie K. Murphy, Alex McConnachie, Cerysh Sadiq, Fozia Haider, Anna Matthews, Laura A. V. Marlow, Kathryn A. Robb

**Affiliations:** 1 School of Medicine, Faculty of Health Sciences and Wellbeing, University of Sunderland, Sunderland, United Kingdom; 2 The Medical College of Wisconsin, Milwaukee, WI, United States; 3 The Initiative on Islam and Medicine, Glendale Heights, IL, United States; 4 Robertson Centre for Biostatistics, School of Health & Wellbeing, University of Glasgow, Glasgow, United Kingdom; 5 School of Medicine, Dentistry & Nursing, University of Glasgow, Glasgow, United Kingdom; 6 School of Health & Wellbeing, University of Glasgow, Glasgow, United Kingdom; 7 Wolfson Institute of Population Health, Queen Mary University of London, London, United Kingdom

**Keywords:** cancer screening, community-based participatory research, community-centred, cultural tailoring, faith-based intervention, health inequalities, Muslim women, religious tailoring

## Abstract

**Introduction:**

Cancer screening saves lives, but Muslim women are less likely to participate in screening than the general population. This makes them less likely to benefit from earlier diagnosis. Faith-based cancer communications provide an acceptable strategy for addressing screening barriers. Hence, we evaluated the impact of a faith-based intervention on knowledge, attitudes, and breast, cervical and colorectal (bowel) screening practices of Muslim women in the UK. Our intervention was a co-designed, peer-led, two-hour workshop informed by the Integrated Screening Action Model (I-SAM) and 3R religiously-tailored messaging model, included peer-led discussions about cancer screening, GP-led education on screening, testimonials from Muslim women, and a female religious-scholar led didactic on Islamic perspectives on screening.

**Methods:**

In 2024, as part of a non-randomised feasibility and efficacy trial, the workshop was co-delivered 16 times, face-to-face or online, with 169 Muslim women in Glasgow and North-East England who were not up-to-date with at least one screening programme. Consistent with I-SAM, outcomes included screening knowledge, attitudes, intentions and self-reported uptake measured at baseline, post-intervention, and 6-and 12-months. Changes from baseline were evaluated with paired tests. Linear regression was used to assess associations with socio-demographic and attitudinal covariates.

**Results:**

Participants were aged 25–74 (mean 47), and 128 (76.6%) identified as South Asian. Mean cancer knowledge more than doubled from pre-intervention to 12-month follow-up (from 3.2/14 to 7.4/14; mean change = 4.2 [95% CI 3.6, 4.8]). Some attitudes changed significantly (e.g., risk perception), although others did not (e.g., worry about male providers). Intention to engage in screening increased significantly from pre-intervention to 12-months for breast (55 to 92%, change = 37% [22, 52%], *n* = 38), cervical (43 to 83%, change = 40% [29, 51%], *n* = 100), and colorectal (35 to 85%, change = 50% [28, 72%], *n* = 20). Among participants invited for screening during 12 months-follow-up, self-reported uptake was 70% (*n* = 14/22) for breast screening, 87% (*n* = 41/50) for cervical screening and 91% (*n* = 10/11) for colorectal screening.

**Conclusion:**

This is the first evaluation of a peer-led, faith-based intervention for British Muslim women and suggests it may be effective in improving screening knowledge, attitudes and behaviour. Further research is needed to determine intervention effectiveness in larger and more robust studies.

## Introduction

Screening provides early detection of breast, colorectal (bowel), and cervical cancer and thus is proven to reduce disease-related morbidity and mortality (1, 2). However, ethnic minority groups and people living in more socioeconomically disadvantaged communities continue to experience inequalities across the cancer pathway, including screening uptake, stage at diagnosis and cancer outcome (3); addressing these inequities has been identified as a key priority in the UK Cancer Strategies (3). A third of the ethnic minority population within the UK is Muslim, and Islam is the second largest religion (4). Accordingly, there are almost 4 million Muslims in the UK, and they form an ethnically diverse population whose religion strongly impacts their health beliefs and behaviours (4, 5). Most British Muslims belong to ethnic minority groups and 40% live in the most deprived quintile (20%) of the Index of Multiple Deprivation (4), suggesting that Muslim communities may be disproportionally affected by ethnic and socioeconomic inequalities observed across the cancer pathway.

The UK has national, organised population screening programmes for breast cancer (ages 50–71, every three years), cervical cancer (ages 25–64, every five years), and colorectal cancer (every two years, ages 50–74 in Scotland and England, with the starting age in England recently lowered from age 60 to 50). Women from ethnic minority backgrounds are less likely to participate in breast, colorectal, and cervical screening than white-British women (6–10). UK South-Asian populations have significantly lower rates of cervical, colorectal and breast screening, again particularly among Muslims (9–11). This is of particular concern for breast screening because Pakistani and Indian Muslim women are at highest risk of breast cancer compared to all other South-Asian ethnic subgroups (12). Early detection through screening is therefore especially important for Muslim women.

Cancer screening is a complex behaviour with multiple barriers and facilitators including individual, social, environmental, and economic factors (13, 14). The multifactorial, dynamic, and inter-relational nature of these barriers indicates that interventions to increase uptake should be complex (15–17). Population-level interventions may be insufficient to address barriers experienced by underserved communities and evidence suggests that interventions tailored to the needs of underserved or specific groups may be more effective than one-size-fits-all approaches (18–22). Interventions that positively influence Muslim people’s attitudes and intention towards breast, colorectal and cervical screening have typically included three components: (i) religiously tailored messages; (ii) peer-education; and (iii) community co-design (23–26). Religious beliefs can influence health behaviour and therefore, faith-based messages are a culturally acceptable approach of addressing screening barriers. These components are consistent with whole-systems approaches to tackling health inequities (27, 28) and in line with the Medical Research Council’s (MRC) guidance on the development and evaluation of complex interventions, which emphasises community involvement in intervention design and delivery (29). We used Community-Based Participatory Research (CBPR) and worked in partnership with Muslim women to co-design an intervention aimed at supporting informed decision-making regarding participation in breast, cervical and colorectal screening. We aimed to investigate whether the intervention could improve knowledge of cancer and its screening, reduce barriers beliefs and improve attitudes towards screening, increase intentions to participate in screening, and increase self-reported uptake of breast, cervical and colorectal screening.

## Methods

### Intervention

In 2021, we co-designed a pilot, peer-led, faith-based intervention to increase breast, colorectal, and cervical screening uptake with Muslim women in Glasgow (n = 10) (30, 31). Intervention development was underpinned by the Behaviour Change Wheel (32) and the Integrated Screening Action Model (I-SAM) (13). The I-SAM was developed to improve understanding of screening behaviour and integrates the stages of behaviour change as described in the Precaution Adoption Process Model (33), targets for behaviour change of the Capability, Opportunity, Motivation- Behaviour (COM-B) model (32), and the interrelationship between individual, social and environmental factors reflected in the socio-ecological model (34). Padela’s 3R ‘Reframe, Reprioritise, and Reform’ model was used to address myths and barriers to screening and develop faith-based messages that encourage screening (35). Details of intervention development, including the faith-based messages, are presented elsewhere (30, 31).

The intervention was composed of a four-part multidimensional intervention. This was a two-hour workshop consisting of (1) peer-led discussions about cancer screening; (2) a health education session delivered by a female GP followed by a question and answer session; (3) videos with personal testimonials of Muslim women sharing experiences with cancer and screening; and (4) an Islamic perspective on screening delivered by a female religious scholar (Figure 1). While the intervention was designed to improve access and reduce barriers to cancer screening for Muslim women, the intervention supported informed choice.

**Figure 1:**
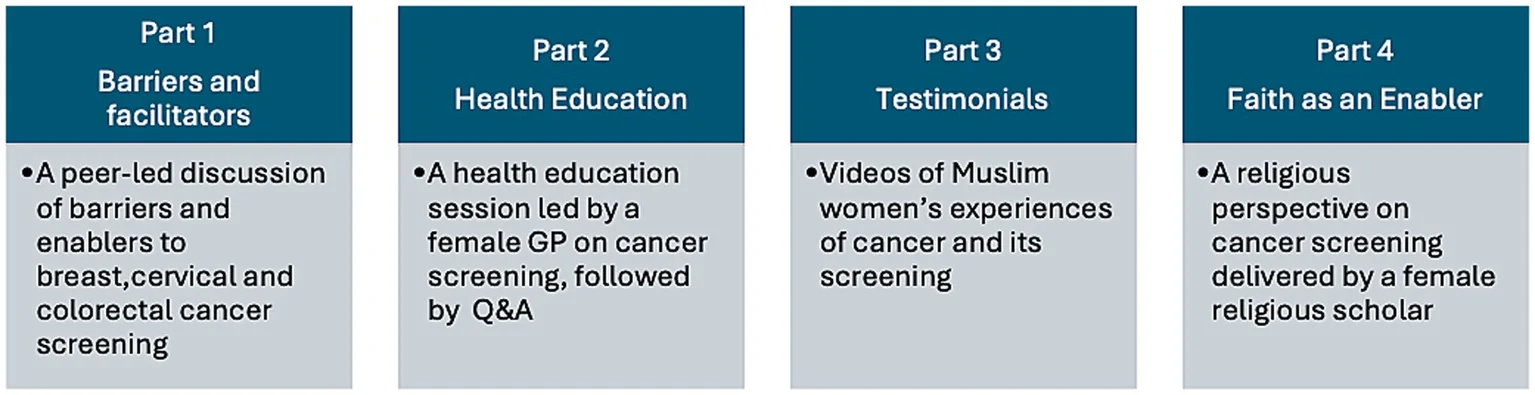
Co-designed, peer-led, faith-based intervention workshop (2 h) to encourage breast, cervical and colorectal screening uptake among Muslim women (30, 31).

A pilot form of the intervention was delivered twice online in 2021 with Scottish Muslim women of Asian and Arab ethnicity (*n* = 18, aged 25–54). Evaluation data revealed high acceptability, increased knowledge and more positive attitudes towards screening among participants, as described elsewhere (30, 31).

### Study design

Building from that pilot, the current study describes the next phase of the work, a non-randomised feasibility and efficacy trial involving two intervention delivery modes (online and face-to-face) with follow-up at six and 12 months and assessment using a pre-post survey design. We anticipated, in line with the I-SAM, that the workshop intervention would increase knowledge about cancer and National Health Service (NHS) cancer screening programmes and reduce barrier beliefs. These changes were expected to increase intention and self-reported uptake of cancer screening following invitation.

### Setting

This study was conducted in two socio-economically deprived areas with generally poor cancer outcomes: Scotland and the North East of England UK (36). Workshops were delivered in mosques or community centres.

### Sample and sampling approach

The current study employed two Community Recruitment Leads (CS and FH), who were members of their local Muslim community, with strong local networks which facilitated participant recruitment. Convenience sampling was used and participants were recruited from Muslim communities and mosques in both locations by advertising the study through their local networks, social media and several community locations. Community organisations that work with Muslim women also helped with the recruitment in some instances. Additional snowball sampling was used to gain a robust sample. We aimed to recruit 50 participants to each mode of delivery and at each site, 200 in total, given that sample sizes between 24 and 50 are recommended for feasibility-type studies (37).

Participants were eligible for the study if they identified as Muslim and lived in the North East of England or Scotland, were aged 25–74 years, and were not up to date with at least one of the breast, cervical or colorectal screening programme for which they were eligible.

Eligibility was initially assessed using a registration form which participants were asked to complete before attending the workshop. Participants were asked whether they were up-to-date with breast, cervical, and colorectal screening based on the recommended screening intervals. However, the survey also asked whether participants had attended screening following their most recent invitation. The latter was considered a more suitable measure of participation in the NHS screening programmes. Therefore, where a discrepancy was found between registration and survey responses, the survey data were used, which resulted in the exclusion of 65 participants at the point of analysis. There were also 22 women who attended the workshop, but who chose not to participate in the study. As these women presented on the day of the workshop, they were accepted into the workshop, but did not complete the surveys.

### Measures

To assess the intervention’s impact, survey data were collected at baseline, post-intervention, 6-and 12-months follow up, focusing on changes to knowledge, barrier beliefs and attitudes, intention to participate in screening, and self-reported breast, cervical and colorectal screening. Survey items were drawn from our previous work, the literature and adapted from existing validated questionnaires where possible. Survey items were reviewed by our Public Involvement and Community Engagement (PICE) group.

The baseline survey captured demographic questions on age, ethnicity, language spoken at home and education. Ethnicity was collected using 17 categories however, because of the small categories other than Asian, these were combined into broader groups for statistical analysis. The sample’s full ethnic distribution is presented in Supplementary material 3.

Psychological acculturation refers to changes in individual behaviours, attitudes, values, and identities that occur through sustained contact with different social environments and value systems. Acculturation has been linked to a variety of health behaviours from conceptualization of illness to help-seeking (38, 39). In this study, demographic proxies for sustained engagement with British culture were used to assess acculturation and an acculturation score (out of 2) was calculated as a weighted combination of age (in years) and home language, as follows: Acculturation score = (Age - 25)/50 + Home_language, where Home_language = 1 if the participant’s home language was “English,” or 0 otherwise (40). Highest acculturation scores therefore indicate the oldest participants whose home language is English.

In the baseline, post-workshop and follow-up surveys, we included the same questions on knowledge of cancer and screening, screening attitudes, and behaviours, which were derived from the Cancer Awareness Measure (CAM) (41). Knowledge questions included breast, cervical or colorectal cancer warning signs, likelihood of each cancer, risk factors, and NHS screening programmes. Knowledge scores were calculated for each type of cancer, assigning a score of 1 or 0 to each item, with the sum giving a total score of 6 for breast, 5 for cervical, and 3 for colorectal. A total cancer knowledge score was calculated as the sum of breast, cervical and colorectal cancer knowledge scores (Supplementary material 1).

To measure attitudes towards screening, we assessed 22 barrier beliefs derived from the CAM (41) and mapped onto the I-SAM (13). The total barrier beliefs score was calculated from 16 of the 22 barrier belief statements (Supplementary material 2), measured using a 4-point Likert scale. We summarised data on all 22 barrier beliefs. However, in the calculation of the total score we excluded six barrier beliefs as these were not specifically addressed in the workshop and our workshop teaching was not related to changing those beliefs; this is consistent with the 3R model. For example, difficulties making an appointment may reflect structural barriers from within the UK healthcare system and we did not believe that our educational workshop could change beliefs about these.

The mean value was calculated and rescaled to have a range of 0 to 100, with higher scores representing greater perceived barriers. Missing values on any of the 16 statements were ignored in the calculation of the mean, though if more than 8 out of the 16 items were missing, the total barrier beliefs score was indicated as missing.

The baseline survey included the Religious Health Fatalism Questionnaire for Muslim populations (RHFQ-M), containing eight statements and measuring agreement to those statements using a 5-point Likert-type scale. This survey had been validated by us and religious health fatalism was found to be associated with Muslim cancer screening behaviour (42). Responses were summarised by item and as a simple summed score on two subscales: the Destined Plan subscale (score 3–15), which consists of 3 items measuring belief that an individual’s health status is part of a plan determined by God, and the Divine Provision subscale (score 5–25), which consists of 5 items measuring belief that God will provide good health (42).

Baseline also included the Modesty Measure for Muslim Women (MMM-W), which assessed participants’ responses to 10 statements about how they apply modesty in their daily lives, measured along a 4-point Likert-type agreement scale, yielding a summed score from 10 to 40 with 40 being the most modest (43).

At baseline, we also asked participants to self-assess their health status using the EuroQol 5-Dimension 5-Level questionnaire; health utility scores were derived using the EQ-5D-5L UK Crosswalk value set (44). The EQ-5D-5L consists of two parts: the descriptive system (EQ-5D) and the EQ visual analogue scale (EQ VAS). The descriptive system comprises five dimensions: mobility, self-care, usual activities, pain/discomfort and anxiety/depression. Each dimension has 5 levels: no problems, slight problems, moderate problems, severe problems and extreme problems, and uses a scale of zero to 100, with zero representing the least healthy and 100 the most healthy (45, 46). The EQ VAS records the patient’s self-rated health on a vertical visual analogue scale (−0.59–1.00) where the endpoints are labelled as ‘The best health you can imagine’ and ‘The worst health you can imagine’ (45, 46).

Baseline (pre-workshop), post-workshop and follow-up surveys assessed changes in participants’ intention to participate in breast, cervical and/or colorectal screening from baseline to post-workshop, and 6-and 12-month follow-up. It also captured self-reported uptake of breast, cervical and colorectal screening at 6 and 12 months.

### Data collection

Between February and June 2024, the intervention was delivered 16 times, ten online workshops and 6 in-person workshops. The survey was administered online using Qualtrics as well as on paper, depending on participants’ preference, and participants could complete the survey in English, or using professionally translated versions in Arabic, Urdu or Bengali.

### Data analysis

Data were cleaned and prepared for analysis, including checks for duplicate entries and invalid responses. Missing data were excluded from relevant analyses. All data analysis was performed in R version 4.4.2 (47). Numerical variables were summarised using the mean and standard deviation. Categorical variables were summarised with number of observations and percentages of participants in each category. Absolute mean changes over time (for continuous measures and for percentages) are reported with 95% confidence intervals. All confidence intervals are presented with associated *p*-values. In this exploratory analysis, these *p*-values are intended as descriptive aids as to which changes may be important (e.g., where *p* < 0.05), but they are not adjusted for multiple comparisons and should be interpreted with caution.

#### Knowledge

Baseline cancer knowledge scores were compared to post-workshop, 6-month follow-up and 12-month follow-up scores using two-sided paired t-tests, without assuming equal variances, under the null hypothesis of no difference between baseline and follow-up time point. Observed differences were summarised with 95% confidence intervals.

#### Barrier beliefs

Changes in the percentage of participants who responded “Agree” or “Strongly agree” to each barrier belief statement from baseline to post-workshop were summarised as proportions and 95% confidence intervals. Statistical significance of these changes was evaluated with z-tests for paired proportions (48). Simple linear regression models were used to determine associations between total barrier belief scores and each of the following predictors: location, workshop mode, age, education, acculturation score, baseline barrier beliefs score, RHFQ-M score, MMM-W score, and change in cancer knowledge score from pre- to post-workshop. Predictors with small *p*-values (*p* < 0.1) from the simple regression models were combined in a multiple linear regression model. Baseline barrier beliefs score was included as covariate to account for participants with higher baseline scores having had more room for decreasing their scores post-workshop.

#### Intention

Absolute changes in the percentage of participants intending to screen for breast, colorectal and cervical cancers, from baseline to post-workshop, 6-month follow-up, and 12-month follow-up time points, were summarised with 95% confidence intervals. Positive intention to screen was defined as responding “Yes, definitely” or “Yes, probably” to intention to go for breast, colorectal and/or cervical cancer screening at the next invitation from the NHS. Statistical significance of the observed changes was evaluated with z-tests for paired proportions. Simple logistic regression models of improvement in intent to screen for cervical cancer, from baseline to post-workshop, were fitted to data from all participants eligible but not up-to-date for cervical cancer screening, who completed both the pre- and post-workshop surveys, and who at baseline were not intending to screen for cervical cancer at the next invitation. Predictors considered in these simple logistic regression models were: location, workshop mode, age, education, acculturation score, RHFQ-M score, MMM-W score, change in cancer knowledge score from pre- to post-workshop, and change in barrier beliefs score from pre- to post-workshop. Predictors with small *p*-values from the simple regression models were combined in a multiple logistic regression model.

#### Self-reported uptake

Proportions of participants eligible for breast, colorectal and/or cervical screening respectively, who had been invited to screening for each of these three cancer types by 12 months follow-up, were calculated. Self-reported uptake for each screening type was calculated from those that received an invitation by 12 months. Simple logistic regression models of self-reported uptake of cervical screening at 12-months follow-up were fitted to data from all participants eligible but not up-to-date for cervical screening, who had been invited for cervical screening between the workshop and 12-month follow-up time points. Predictors with small *p*-values (*p* < 0.1) in the simple regression models were included together in a multiple logistic regression model. Similar analyses of self-reported uptake for breast and bowel cancer screening were not feasible due to insufficient sample sizes.

### Patient and public involvement

The IMCAN study was grounded in principles of Community-Based Participatory Research with the involvement of community leaders and members at each stage of the study from conception to dissemination (27, 49, 50). The original intervention was co-designed with 10 Muslim women, including a female religious leader (30, 31). Building on this pilot work, we involved members of the community in the IMCAN project from the development of the funding application onwards. Once the project started, two community members (CS and FH) were employed by the project as Community Recruitment Leads, facilitating relationship building and participant recruitment. We also trained 15 women as ‘peer facilitators’ to support intervention delivery. The project was further supported by a dedicated Public Involvement Community Engagement (PICE) group, which met on average four times per year and advised the project at every step. This included reviewing the data collection measure, contributing to qualitative data analysis through collaborative transcript coding and discussion of emerging themes, and interpretation of findings and dissemination. Findings were shared with the community in two dissemination events in February 2026, for which we co-created accessible materials with the PICE group, including an animation, a two-page visual summary of the study and a bookmark.

## Results

In total 256 women attended the IMCAN workshops, of whom 234 (91.4%) consented to taking part in the study and completed the baseline survey. Of those completing the survey, 65 (27.8%) were excluded from the study due to being up-to-date with all previous cancer screenings they were eligible for, leaving *n* = 169 (72.2%) participants eligible for the study and to be included in the analysis. Of eligible participants, 52 (30.8%) were not-up-to date with breast screening, 148 (87.6%) were not up to date with cervical screening, and 22 (13.0%) were not up-to-date with colorectal screening (Figure 2).

**Figure 2:**
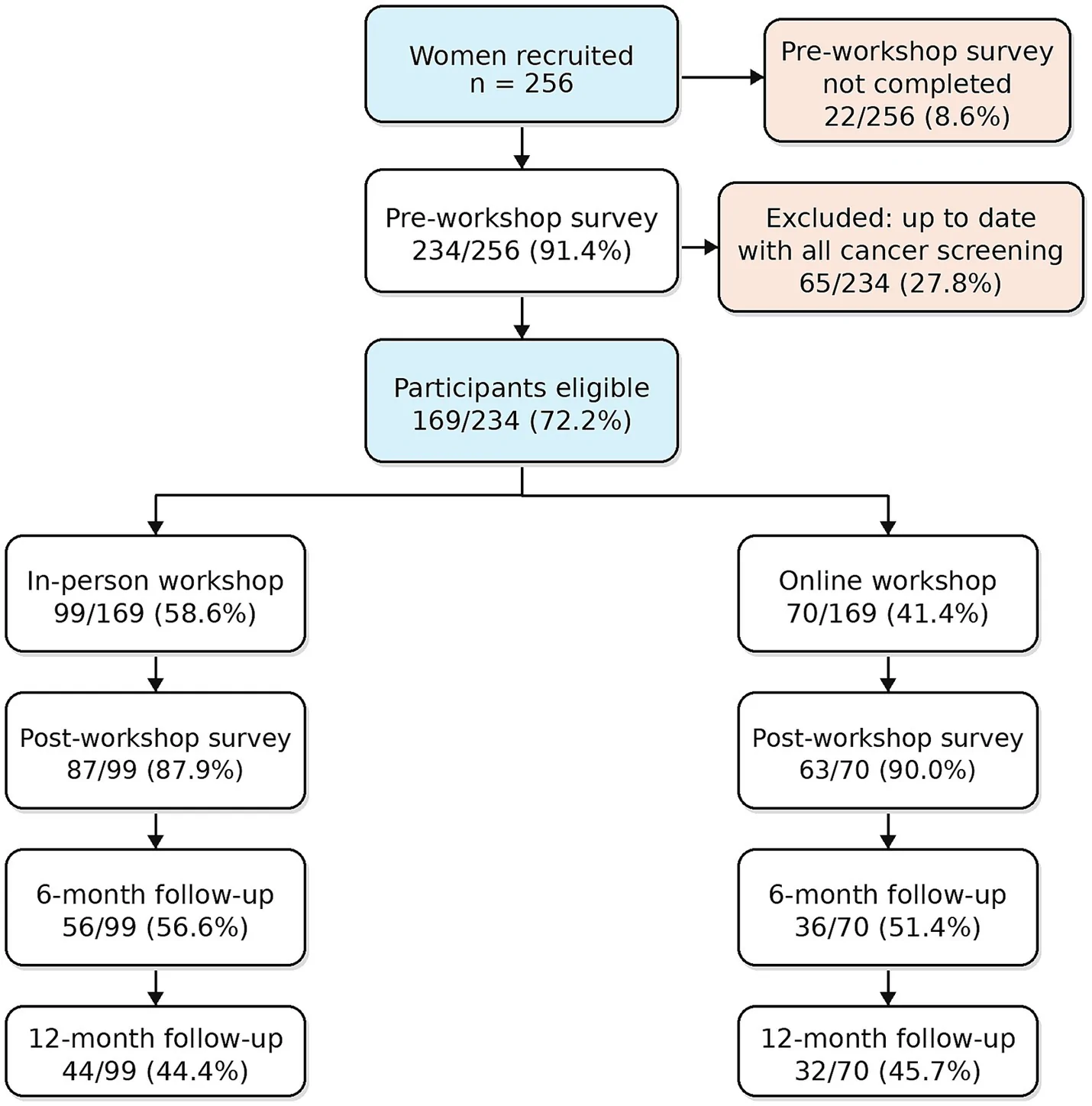
Participant flowchart.

Of the 169 participants, 92 were recruited in England and 77 in Scotland (Table 1). The mean age of participants was 47.0 years (SD = 13.6), with participants in Scotland being older on average than those in England (49.9 vs. 44.5 years). Overall, 59.2% (*n* = 100) of participants were aged under 50 years, although there was a higher proportion of older participants (≥60 years) in Scotland compared to England (32.5% (*n* = 25) vs. 12.0% (*n* = 11)). The sample was predominantly of Asian ethnicity (76.6%, *n* = 128), with a higher proportion of participants Asian in Scotland (86.7%, *n* = 65) than in England (68.5%, *n* = 63).

**Table 1:** Participant characteristics.

Variable	Statistic	All (*n* = 169)	England (*n* = 92)	Scotland (*n* = 77)
Age (*n* = 169)	Mean in years (SD)	47.0 (13.6)	44.5 (12.0)	49.9 (14.9)
	Range	(25.0, 74.0)	(25.0, 74.0)	(26.0, 74.0)
Age group				
25–49	N (%)	100 (59.2)	63 (68.5)	37 (48.1)
50–59	N (%)	33 (19.5)	18 (19.6)	15 (19.5)
60–75	N (%)	36 (21.3)	11 (12.0)	25 (32.5)
Ethnicity (*n* = 167)				
Asian	N (%)	128 (76.6)	63 (68.5)	65 (86.7)
Black	N (%)	5 (3.0)	4 (4.3)	1 (1.3)
White	N (%)	3 (1.8)	0 (0.0)	3 (4.0)
Other	N (%)	31 (18.6)	25 (27.2)	6 (8.0)
Home language (*n* = 169)				
Arabic	N (%)	16 (9.5)	14 (15.2)	2 (2.6)
Bangla/Bengali	N (%)	26 (15.4)	26 (28.3)	0 (0.0)
English	N (%)	48 (28.4)	20 (21.7)	28 (36.4)
Punjabi	N (%)	17 (10.1)	9 (9.8)	8 (10.4)
Urdu	N (%)	51 (30.2)	14 (15.2)	37 (48.1)
Other	N (%)	11 (6.5)	9 (9.8)	2 (2.6)
Education (*n* = 164)				
No formal qualifications	N (%)	50 (30.5)	28 (31.1)	22 (29.7)
Secondary education	N (%)	29 (17.7)	19 (21.1)	10 (13.5)
Higher education (post-secondary qualifications below degree level)	N (%)	18 (11.0)	6 (6.7)	12 (16.2)
Degree (undergraduate or postgraduate)	N (%)	41 (25.0)	20 (22.2)	21 (28.4)
Other/do not know/prefer not to say	N (%)	26 (15.9)	17 (18.9)	9 (12.2)
Acculturation score (*n* = 169)	Mean on scale 0 to 2 (SD)	0.7 (0.5)	0.6 (0.4)	0.9 (0.5)
	Range	(0.0, 1.9)	(0.0, 1.8)	(0.0, 1.9)
EQ-5D-5L index score (n = 156)	Mean on scale −0.59 to 1.00 (SD)	0.76 (0.23)	0.77 (0.25)	0.76 (0.21)
	Range	(−0.08, 1.00)	(−0.08, 1.00)	(0.01, 1.00)
EQ Visual analogue scale^*^ (*n* = 159)	Mean on scale 0 to 100 (SD)	69.3 (18.4)	69.5 (17.0)	69.0 (20.1)
	Range	(0.0, 100.0)	(30.0, 100.0)	(0.0, 99.0)
MMM-W** total score (*n* = 125)	Mean on scale 8 to 40 (SD)	31.7 (5.2)	32.3 (5.0)	31.0 (5.3)
	Range	(10.0, 40.0)	(22.0, 40.0)	(10.0, 40.0)
Total RHFQ-M*** score (*n* = 128)	Mean on scale 8 to 40 (SD)	30.5 (8.7)	32.1 (7.6)	28.4 (9.6)
Destined plan subscale (*n* = 135)	Mean on scale 3 to 15 (SD)	11.3 (3.4)	11.8 (3.3)	10.8 (3.5)
Divine provision subscale (*n* = 131)	Mean on scale 5 to 25 (SD)	19.1 (5.7)	20.3 (4.8)	17.8 (6.4)

*EuroQol Visual analogue scale.

** Modesty Measure for Muslim Women (MMM-W).

*** Religious Health Fatalism Questionnaire for Muslim populations (RHFQ-M).

Participants reported a wide range of home languages. The most commonly reported languages were Urdu (30.2%, *n* = 51), English (28.4%, *n* = 48), and Bengali or Bangla (15.4%, *n* = 26). Some regional differences were observed, with Urdu more commonly spoken in Scotland (48.1%) and Bangla and Bengali more prevalent in England (28.3%).

Educational attainment varied across the sample. Approximately 30.5% (*n* = 50) of participants reported no formal qualifications, while 25.0% (*n* = 41) held a degree.

Acculturation scores on a scale of 0 to 2 were generally low (mean = 0.7, SD = 0.5), with slightly higher scores in Scotland (mean = 0.9) compared to England (mean = 0.6).

Health status, measured using the EQ-5D-5L index, was relatively high across the sample (mean = 0.76, SD = 0.23), with similar scores observed between England and Scotland.

Mean scores for the RHFQ-M were 30.5 (SD = 8.7), with higher scores in England (32.1) than in Scotland (28.4). MMM-W scores were similar across groups, with an overall mean of 31.7 (SD = 5.2).

### Knowledge

Table 2 shows the change in cancer knowledge scores from baseline to post-workshop, 6-month, and 12-month follow-up surveys, respectively. Significant increases in cancer knowledge scores were observed from baseline to post-workshop time points for all three cancer types and overall, and these increases persisted at the 6- and 12-month follow-up.

**Table 2:** Mean change in cancer knowledge scores between baseline and follow-up.

Comparison	Baseline	Follow-up (FU)	Change	95% CI	*p*-value
Breast cancer knowledge score (0–6)					
Baseline to Post-workshop (*n* = 169)	1.5	3.2	+1.7	(+1.4; +2.0)	<0.001
Baseline to 6-month FU (*n* = 103)	1.6	3.6	+2.0	(+1.6; +2.3)	<0.001
Baseline to 12-month FU (*n* = 118)	1.6	3.4	+1.8	(+1.5; +2.2)	<0.001
Cervical cancer knowledge score (0–5)					
Baseline to Post-workshop (*n* = 169)	1.0	2.8	+1.8	(+1.5; +2.0)	<0.001
Baseline to 6-month FU (*n* = 103)	1.2	2.6	+1.5	(+1.2; +1.8)	<0.001
Baseline to 12-month FU (*n* = 118)	1.1	2.7	+1.7	(+1.4; +1.9)	<0.001
Colorectal cancer knowledge score (0–3)					
Baseline to Post-workshop (*n* = 169)	0.6	1.3	+0.7	(+0.6; +0.9)	<0.001
Baseline to 6-month FU (*n* = 103)	0.6	1.4	+0.8	(+0.6; +0.9)	<0.001
Baseline to 12-month FU (*n* = 118)	0.6	1.3	+0.7	(+0.5; +0.9)	<0.001
Total cancer knowledge score (0–14)					
Baseline to Post-workshop (*n* = 169)	3.1	7.3	+4.2	(+3.6; +4.8)	<0.001
Baseline to 6-month FU (*n* = 103)	3.3	7.6	+4.2	(+3.6; +4.9)	<0.001
Baseline to 12-month FU (*n* = 118)	3.2	7.4	+4.2	(+3.6; +4.8)	<0.001

### Barriers to screening

Table 3 shows the change in barriers to screening from baseline to post-workshop, for participants (*n* = 76) who completed all four surveys (baseline, post-workshop, 6-month follow-up, 12-month follow-up).

**Table 3:** Change in barriers to screening from baseline to post-workshop.

Barrier (I-SAM* domain)	n	Baseline	Post-workshop	Change	95% CI	*p*-value
Reflective motivation						
Breast, colorectal or cervical cancer screening is not needed without symptoms	65	43%	22%	−22%	(−35%; −8%)	0.004
I am not at risk of developing colorectal cancer	62	39%	23%	−16%	(−30%; −2%)	0.033
I do not think that I am at risk of cervical cancer	59	36%	22%	−14%	(−27%; +0%)	0.059
I have had a bad experience of cancer screening in the past	57	28%	25%	−4%	(−19%; +12%)	0.655
The harms of cancer screening outweigh the benefits	54	30%	30%	0%	(−18%; +18%)	1.000
Automatic motivation						
Breast screening might be painful	66	56%	47%	−9%	(−25%; +6%)	0.257
Colorectal screening is too messy	60	67%	38%	−28%	(−46%; −11%)	0.003
Cervical screening might be painful	55	67%	67%	0%	(−15%; +15%)	1.000
I am too embarrassed to go for cancer screening	59	53%	39%	−14%	(−27%; +0%)	0.059
I am too frightened of what a cancer screening test might find	56	70%	55%	−14%	(−28%; −1%)	0.046
I am worried about putting extra strain on the NHS/health services	55	33%	29%	−4%	(−17%; +10%)	0.593
Physical opportunity						
I find it difficult to get an appointment	55	62%	42%	−20%	(−33%; −7%)	0.005
It is difficult to get an appointment at a convenient time	57	67%	53%	−14%	(−27%; −1%)	0.046
The screening appointment is too far away from my home	57	32%	21%	−11%	(−23%; +2%)	0.109
Capability						
I find it too difficult to complete the colorectal screening test kit	55	55%	35%	−20%	(−35%; −5%)	0.016
I have other more important things to worry about than cancer screening	63	40%	16%	−24%	(−37%; −11%)	0.001
I am too busy for cancer screening	56	34%	20%	−14%	(−27%; −2%)	0.033
I cannot afford to cover the costs of doing cancer screening	56	32%	25%	−7%	(−19%; +5%)	0.248
I worry my pay/earnings would be affected if I needed to have further tests or treatment	56	34%	30%	−4%	(−17%; +9%)	0.593
Social opportunity						
I am worried about having to take my clothes off or that too much skin would show	57	60%	53%	−7%	(−21%; +7%)	0.346
I do not want a man to carry out the screening test	60	80%	87%	+7%	(−5%; +19%)	0.285
Facilitator						
Nothing would put me off a cancer screening test	56	45%	59%	+14%	(−2%; +30%)	0.088

* Integrated Screening Action Model (I-SAM).

From baseline to post-workshop, agreement with several barrier beliefs significantly declined (Table 3). Significant reductions were observed for barriers related to reflective and automatic motivation, including the beliefs that “Breast, colorectal or cervical cancer screening is not needed without symptoms” (−22%; *p* = 0.004), “I am not at risk of developing colorectal cancer” (−16%; *p* = 0.033), “Colorectal screening is too messy” (−28%; *p* = 0.003), and “I am too frightened of what a cancer screening test might find” (−14%; *p* = 0.046).

Significant reductions were also observed for barriers related to capability, including “I find it too difficult to complete the colorectal screening test kit” (−20%; *p* = 0.016), “I have other more important things to worry about than cancer screening” (−24%; *p* = 0.001), and “I am too busy for cancer screening” (−14%; *p* = 0.033).

There were also significant decreases observed in physical opportunity related to appointment barriers, “I find it difficult to get an appointment” (−20%; *p* = 0.005), and “It is difficult to get an appointment at a convenient time” (−14%; *p* = 0.046).

Little change was observed for several barrier beliefs, including social opportunity barriers relating to concerns about modesty during screening and preference for a female healthcare provider; reflective motivation barriers concerns that breast or cervical screening might be painful and potentially harmful; and the physical opportunity barrier relating to financial costs.

In simple logistic regression models, decrease in barrier beliefs from baseline to post-workshop was associated with higher baseline barrier beliefs score (*p* < 0.001), lower education level (*p* < 0.001), and increase in cancer knowledge score from pre- to post-workshop (*p* < 0.001). Change in barrier beliefs was not associated with location (*p* = 0.23), workshop mode (*p* = 0.76), age (*p* = 0.50), acculturation score (*p* = 0.17), RHFQ-M score (*p* = 0.60), or MMM-W score (*p* = 0.96). In the multiple regression model (Table 4), a decrease in barrier beliefs score was associated with higher baseline barrier beliefs score (*p* < 0.001) and an increase in cancer knowledge from pre- to post-workshop (*p* < 0.001) but not with education level (*p* = 0.2).

**Table 4:** Multiple linear regression model.

Predictor	Estimate [95% CI]	*p*-value
Education (ref: None)		0.200
Secondary	−7.66 [−15.95; 0.62]	
Higher education	−4.85 [−13.45; 3.75]	
Degree	1.67 [−5.78; 9.12]	
Other	−2.55 [−11.32; 6.23]	
Baseline barrier score (polynomial)		<0.001
Baseline barrier score	0.16 [−0.58; 0.89]	
Baseline barrier score^2^	−0.01 [−0.01; 0.00]	
Cancer knowledge change (pre- to post-workshop)	−1.61 [−2.41; −0.81]	<0.001

### Intention to screen

For breast cancer screening, prior to the workshop, 28 participants (57% of those eligible but not up-to-date for breast cancer screening) intended to participate in breast screening at the next invitation (Table 5). Intention to screen increased significantly from pre- to post-workshop (+34%; *p* < 0.001) and persisted at 6 months (+24%; *p* = 0.011) and 12 months (+37%; *p* < 0.001).

**Table 5:** Change in intention to participate in breast, cervical and colorectal screening.

Comparison	Baseline	Follow-up (FU)	Change	95% CI	*p*-value
Breast					
Baseline to Post-workshop (*n* = 41)	59%	93%	+34%	(+18%; +50%)	<0.001
Baseline to 6-month FU (*n* = 34)	56%	79%	+24%	(+7%; +40%)	0.011
Baseline to 12-month FU (*n* = 38)	55%	92%	+37%	(+22%; +52%)	<0.001
Colorectal					
Baseline to Post-workshop (*n* = 15)	40%	93%	+53%	(+28%; +79%)	0.005
Baseline to 6-month FU (*n* = 14)	50%	93%	+43%	(+17%; +69%)	0.014
Baseline to 12-month FU (*n* = 20)	35%	85%	+50%	(+28%; +72%)	0.002
Cervical					
Baseline to Post-workshop (*n* = 129)	50%	91%	+41%	(+32%; +50%)	<0.001
Baseline to 6-month FU (*n* = 84)	43%	81%	+38%	(+26%; +50%)	<0.001
Baseline to 12-month FU (*n* = 100)	43%	83%	+40%	(+29%; +51%)	<0.001
Self-sampling kit do to at home baseline Pre-to 12-month FU (*n* = 99)	36%	31%			
Cervical screening by nurse/doctor	29%	41%			

Prior to the workshop, eight participants (38% of those eligible but not up-to-date for colorectal screening) intended to take up colorectal screening at the next invitation. Intention to screen increased significantly from pre- to post-workshop (+53%; *p* = 0.005) and persisted at 6 months (+43%; *p* = 0.014) and 12 months (+50%; *p* = 0.002).

Prior to the workshop, 67 participants (46% of those eligible but not up-to-date for cervical screening) intended to take up cervical screening at the next invitation. Intention to screen increased significantly from pre- to post-workshop (+41%; *p* < 0.001) and persisted at 6 months (+38%; *p* < 0.001) and 12 months (+40%; *p* < 0.001). A subset of n=116 eligible participants completed the cervical home screening choice questions on both the pre- and post-workshop surveys. Pre-workshop 31% of these participants preferred Option 2: Cervical screening by nurse/doctor, increasing to 41% in the post-workshop survey. This change of 10% (95% CI: -2%; +21%) is not statistically significant in a test of paired proportions (*p* = 0.101).

In the multiple logistic regression model, improvement in intention to screen for cervical cancer, from pre- to post-workshop, was significantly associated with a lower acculturation score only (OR 0.09, 95% CI 0.00–0.98; *p* = 0.048). Therefore, younger participants whose home language was not English were more likely to show improvement in intention to screen for cervical cancer at next invitation, following the workshop. None of the other variables (education, RHFQ-M score, cancer knowledge change) included in the model, reached statistical significance.

### Self-reported screening uptake

Of the 52 participants eligible but not up-to-date for breast screening at baseline, 40 completed the 12-months survey. Of these, 22 (58%) reported being invited for screening within 12 months following the workshop. Fourteen participants (70% of those invited) reported that they completed breast screening, with a higher proportion in Scotland (79%) compared to North East England (50%).

Of the 22 participants eligible but not up-to-date for colorectal screening, 11 (52%) were invited for screening within 12 months following the workshop. Ten participants (91% of those invited) reported that they completed colorectal screening.

Of the 148 participants eligible but not up-to-date for cervical screening, 50 (55%) were invited for screening within 12 months following the workshop. Forty-one participants (87% of those invited) reported that they completed cervical screening, with a higher proportion in Scotland (96%) compared to North East England (77%) (Table 6).

**Table 6:** Self-reported uptake of breast, colorectal and cervical screening at 12 months.

Variable	Statistic	All	England	Scotland
Breast screening				
Invited to breast cancer screening in past 12 months	N_obs_ (N_miss_)	38 (2)	14 (1)	24 (1)
Yes	N (%)	22 (57.9%)	7 (50.0%)	15 (62.5%)
No	N (%)	16 (42.1%)	7 (50.0%)	9 (37.5%)
Completed breast cancer screening in past 12 months	N_obs_ (N_miss_)	20 (2)	6 (1)	14 (1)
Yes	N (%)	14 (70.0%)	3 (50.0%)	11 (78.6%)
No	N (%)	6 (30.0%)	3 (50.0%)	3 (21.4%)
Colorectal screening				
Invited to colorectal cancer screening in past 12 months	N_obs_ (N_miss_)	21 (0)	5 (0)	16 (0)
Yes	N (%)	11 (52.4%)	1 (20.0%)	10 (62.5%)
No	N (%)	10 (47.6%)	4 (80.0%)	6 (37.5%)
Completed colorectal cancer screening in past 12 months	N_obs_ (N_miss_)	11 (0)	1 (0)	10 (0)
Yes	N (%)	10 (90.9%)	0 (0.0%)	10 (100.0%)
No	N (%)	1 (9.1%)	1 (100.0%)	0 (0.0%)
Cervical screening				
Invited to cervical cancer screening in past 12 months	N_obs_ (N_miss_)	91 (12)	50 (8)	41 (4)
Yes	N (%)	50 (54.9%)	25 (50.0%)	25 (61.0%)
No	N (%)	39 (42.9%)	24 (48.0%)	15 (36.6%)
No, but contacted GP and requested appointment	N (%)	2 (2.2%)	1 (2.0%)	1 (2.4%)
Completed cervical cancer screening in past 12 months	N_obs_ (N_miss_)	47 (3)	22 (3)	25 (0)
Yes	N (%)	41 (87.2%)	17 (77.3%)	24 (96.0%)
No	N (%)	6 (12.8%)	5 (22.7%)	1 (4.0%)

In simple logistic regression models, cervical cancer screening uptake at 12-months follow-up was associated with living in Scotland/NE England (*p* = 0.03), an increase in cancer knowledge score from pre- to post-workshop (*p* = 0.02) and a decrease in barrier beliefs score from pre- to post-workshop (*p* = 0.05), but was not associated with workshop mode (*p* = 0.98), age (*p* = 0.76), education level (*p* = 0.73), acculturation score (*p* = 0.67), RHFQ-M score (*p* = 0.95), or MMM-W score (*p* = 0.36).

In the multiple logistic regression model (Table 7), participants were more likely to report uptake of cervical screening at 12 months follow-up if they were living in Scotland (*p* = 0.01), had increased in cancer knowledge from pre- to post-workshop (*p* < 0.001), or decreased barrier beliefs from pre- to post-workshop (*p* = 0.013).

**Table 7:** Predictors of self-reported cervical screening.

Predictor	OR [95% CI]	*p-*value
Location (ref: North East England)		0.010
Scotland	121.3 [2.36; 294639.52]	
Cancer knowledge change (pre- to post-workshop)	3.23 [1.47; 13.57]	<0.001
Barrier beliefs change (pre- to post-workshop)	0.83 [0.62; 0.97]	0.013

## Discussion

This is the first study to utilise a faith-based, participatory approach to investigate a peer-led, co-designed intervention to encourage the uptake of breast, cervical and colorectal screening among Muslim women. As part of a non-randomised feasibility trial, the two-hour workshop intervention informed by the I-SAM, was delivered 16 times, face-to-face or online, with 169 Muslim women in Glasgow and North-East England who were not up-to-date with at least one screening programme. The impact of the intervention was investigated using pre- and post-workshop surveys, with follow-up at 6 and 12 months, assessing changes in knowledge, attitudes and practices of breast, cervical and colorectal screening, consistent with the I-SAM.

Findings suggest the intervention may be effective in improving knowledge, attitudes, intention and self-reported uptake of screening. The percentage of women intending to engage in screening increased significantly from pre-intervention to 12-month follow-up for all three types of screening. For those who had been invited for screening at 12-month follow-up, self-reported screening uptake was 70% for breast, 87% for cervical, and 91% for colorectal. In comparison, in 2023–2024 national uptake was 70.6% for breast, 68.6% for cervical and between 56.6 and 73.5% for bowel screening (51). As screening uptake is thought to be lower for Muslim women, these preliminary result are encouraging although they should be interpreted with caution because of the small sample, self-reported uptake and lack of control group.

However, knowledge of all three screening types also significantly increased from baseline to post- intervention, with scores approximately doubling, and this improvement was sustained at 6- and 12-months follow-up. The increase in knowledge was also a significant predictor of self-reported uptake of cervical screening. Lack of knowledge of cancer and its screening has been found to be a key barrier to screening uptake, while having some knowledge of cancer has been found to be a facilitator and a prerequisite for screening participation (14, 20, 52). Therefore, most interventions promoting screening, include a health education component and a discussion about the importance of cancer screening (14). Other studies that have focused on Muslim communities, also included a health education component and found significant improvements in knowledge after their intervention (26, 53–55), although none of the studies focused on all three types of cancer screening, and the only study conducted in the UK, focused on colorectal screening alone (26).

In the current study, the health education component was delivered by a female GP. In terms of intervention implementation and scalability, involvement of such a clinician may be difficult to achieve due to workforce pressures for clinicians. Evidence from a scoping review investigating educational interventions to improve colorectal cancer awareness and screening in OECD countries, suggested that both health professional as well as lay community health education showed improvements in screening participation and awareness (56), indicating that effective health education delivery may not have to be clinician-led. A hybrid approach might offer a possible compromise, consisting of intervention delivery with trained peer-facilitators and the clinician live online to offer participants the opportunity to ask questions, or prerecorded health education from a clinician.

The association between increased knowledge and reductions in barrier beliefs suggests that improving knowledge may be one of the ways in which the intervention supports engagement with screening. However, the lack of association with education level suggests that these barriers may not simply be related to educational level and may instead reflect broader cultural and social influences. While improving knowledge is important, it is unlikely to be sufficient on its own to address these broader and more complex barriers.

These findings highlight the need for public health interventions that go beyond health education alone and consider wider cultural, social, and contextual factors that influence cancer screening behaviour. Given the complexity of cancer screening behaviour, interventions must be multifaceted, combining health education with culturally tailored, participatory and community-centred approaches (13, 14).

A key strength and innovation of the study was then also our Community-Based Participatory Research approach, which has been identified as important in ensuring that interventions are culturally safe and relevant (27, 57). In the current study, Muslim women were involved at every step of the research. Muslim women were involved in the co-design, the development of the study’s faith-based component, participant recruitment, intervention delivery, data collection, data analysis, interpretation of findings, and dissemination. Other faith-based studies with this population used some of these elements, for example, the co-design element (23, 24, 55, 58), working with community and religious leaders (23), testimonials (53) or peer delivery (23, 55), yet apart from Padela et al., no studies seemed to include all these participatory elements, and none could be found where a female religious scholar was included as part of the intervention design and implementation team. Women might hesitate to participate in interventions on cancer screening if men are present and gender concordance of intervention delivery may have strengthened our intervention (24, 31, 59, 59).

As Muslim women face barriers to screening that may be uniquely related to their worldview, it is important to have culturally and religiously-tailored messages, which outperform non-tailored messages in promoting cancer screening (19, 20).

In our pilot work (30, 31), we explored key barriers to screening, mapped these to the Behaviour Change Wheel (61) to inform the intervention and utilised the Reframe, Reprioritize and Reform (3R) model (35) to develop, in partnership with Muslim women and the female religious scholar, counteracting faith-based messages. A growing evidence-base is demonstrating that faith-based approaches can be utilised in health promotion (60). Faith-based approaches can be considered a form of asset-based approaches because they leverage existing spiritual, cultural and social strengths within religious communities. Engagement with faith communities is valuable in public health as they are often trusted sources of information and guidance, and including religious leaders in health promotion has been recognised as an important and culturally appropriate vehicle for delivering health information (60, 61). A unique component of this study was the inclusion of a female religious scholar in the workshop’s delivery. Health is central in Islam, which encourages the notion of stewardship and individuals to prioritise their health (62). Involving religious leaders directly in health promotion and cancer screening can help overcome religious misconceptions (62). In addition, in this study, the strong collaboration between the GP and the female religious scholar may have helped women to feel at ease and allowed screening promotion messages to be reinforced. At times, participants would check information received by the GP with the female religious scholar.

Although participants scored relatively high on the religious fatalism scale, which has been found to act as a barrier to cancer screening (52), higher levels of fatalism did not seem to prevent participants from benefitting from the intervention, with significant reductions observed across several barrier beliefs following the workshop. Notions of modesty have been found to hinder Muslims from seeking healthcare, especially when a gender concordant health provider may not be available (59, 63). Muslim women may be reluctant to expose parts of their body or obtain healthcare from a provider of the different sex (52, 63). Participants in the current study had a high aggregate level of modesty, indicating that modesty was an important factor influencing decision making around engagement with screening, which is supported in the existing literature (24, 35, 53, 55, 64). Pre-intervention, most participants stated they did not want a man to conduct the screening test and were worried about having to take off their clothes or about too much skin showing. These barrier beliefs related to modesty and gender concordant care showed little change post-intervention. This is perhaps unsurprising, as the intervention did not seek to change participants’ religious and cultural values. Rather, the GP and female religious scholar provided practical support to overcome these barriers by reassuring women that it was unlikely that a man would carry out the screening test and discussed at length that women are allowed to request a female health care provider within the UK’s National Health Service. The female religious scholar also explained that Islam permits treatment by a male healthcare provider if needed (65). The GP, reinforced by the female religious scholar, also provided practical tips for maintaining modesty during screening, such as bringing a sheet. Practical tips were also provided for colorectal screening such as how to collect a stool sample. Post-intervention, significant improvements were seen in related barrier beliefs ‘Colorectal screening is too messy’ and ‘I find it too difficult to complete the colorectal screening test kit’, suggesting that these practical tips helped. Another study with Muslim people conducted in the UK also provided practical support on how complete the FIT test at home, which also found positive results (26). Emphasising the option of a gender-concordant healthcare provider and offering practical tips to support screening uptake, can be readily implemented within UK screening programmes.

Findings showed that reductions in barrier beliefs were associated with higher baseline barrier scores and increases in cancer knowledge following the workshop. The association with baseline barrier belief scores is likely to reflect that participants with higher initial barriers had more scope for change but also suggests that the intervention was particularly effective among those with the greatest perceived barriers. These findings suggest that many barrier beliefs are modifiable and can be addressed through targeted interventions like ours.

Self-sampling has been proposed as a potential solution for populations who are hesitant about cervical screening (66, 67). The only UK study that explored this option was a qualitative study with 18 participants, who perceived self-sampling as acceptable although more than half would still prefer a clinician-collected sample (68). In the current study, a higher proportion of participants initially preferred self-sampling over cervical screening by nurse/doctor pre-intervention (36% vs. 29.4%), although this had shifted at post-intervention and 12-month follow-up (31.3% vs. 41.4%). This change may reflect increased confidence and comfort with clinician-led screening post-intervention, suggesting that the idea of self-sampling may reduce some barriers, but these barriers may also be reduced by our culturally tailored intervention.

### Limitations and strengths

One limitation of the study was that some women were excluded from data analysis as they did not meet the eligibility criteria, which reduced the overall sample size for particular cancers. However, this also reflects a strength of the study as analyses only included women who were not up-to-date with at least one of the screenings they were eligible for. This ensured that the impact of the intervention was investigated only for women targeted by the intervention, while demonstrating that the intervention can be delivered to communities as a whole without excluding women up-to-date for screening. Although women were only eligible if they were not up to date with their screening, it may be the case that participants differed in terms of willingness to learn about screening to women who chose not to attend the workshop. Future research could explore whether embedding intervention components within existing community or religious activities could also increase engagement with screening among women who are not up to date with their screening and who would not agree to attend a standalone workshop.

Another strength of the study was the diversity of the sample, which included women from a range of age groups, home languages and education backgrounds. Although convenience and snowball sampling seemed to work well, sampling through community and religious networks may have introduced selection bias and women who are less socially connected or less engaged with religious and community structures may have been underrepresented. The diversity in age groups was a result of the intervention addressing breast, cervical and colorectal cancer screening together, whilst many studies focus on one cancer screening type. We took this approach as many barriers and facilitators to screening overlap. Therefore addressing these together in a single intervention offers a pragmatic approach to tackling inequalities in cancer screening. However, as not all participants were eligible for all three screening programmes, analyses relating to each screening programme were based on relatively small sample sizes.

Another limitation of the study was that the follow-up period was too short for all participants to receive invitations for their eligible cancer screenings. Ideally, the follow-up in a definitive trial would extend to five years, which would be long enough for all eligible participants to have been invited to screening. In addition, using self-reported survey data, which may have biased our findings. Previous research comparing self-report to medical records, found that particularly individuals who had not engaged in screening over-reported screening uptake (69). Using NHS screening data would offer a more objective measure of screening attendance following the intervention. However, self-report remains a commonly used method for assessing screening behaviour and may be the most feasible approach in community-based studies. Participants were also aware that the study aimed to improve screening uptake, which may have influenced their reported attitudes, intentions and screening behaviour. They may have perceived an expectation to engage in screening or to provide responses that they considered favourable. Nevertheless, the behavioural changes observed in the study, including the improved intention to engage with screening and the self-reported uptake at 12-month follow-up, as well as the improved knowledge and reduced barrier beliefs, are encouraging and suggest the intervention has the potential to influence screening behaviours. These findings support the need for a future definitive trial with longer follow-up to objectively measure screening uptake. If the intervention’s effectiveness is confirmed in a definitive trial, this could inform partnerships between NHS screening services and Muslim community and faith organisations. Incorporating culturally and religiously tailored interventions may help address barriers to screening and tackle screening inequalities.

In conclusion, this study describes a co-designed and peer-led intervention for Muslim women that used religious messages to support engagement with cancer screening for early diagnosis. Findings suggest the intervention may have the potential to improve knowledge, reduce screening related barriers beliefs, and increase intentions to participate in screening. Although the follow-up period was not sufficiently long for all participants to have been invited to screening, self-reported screening uptake was high for those who reported to have been invited. Religiously-tailored health messages appear to be an effective modality to promote cancer screening. Our vision is to tackle current cancer inequities by driving forward the development of this co-designed faith-based, peer-led intervention to address both knowledge gaps and barriers to accessing screening to improve engagement with breast, colorectal and cervical screening among Muslim women in partnership with their communities and assess the intervention more robustly through a larger-scale randomised evaluation.

## Data Availability

Anonymised data are available from the corresponding author on reasonable request.
