# Supplementary material for: Peer-led, faith-based co-designed intervention to improve Muslim women’s cancer screening: results from the IMCAN study
Source: Front Public Health. 2026 Oct 9;14:1918791. doi: 10.3389/fpubh.2026.1918791 (PMC13690773; doi:10.3389/fpubh.2026.1918791)
Supplement: Supplementary file 1 [file Table_1.DOCX]

**Supplement 1**

**Knowledge scales**

**Breast cancer knowledge score**

Breast cancer knowledge score was calculated from the following items, assigning a score of 1 or 0 to each, with the sum giving the total score (out of 6):

- Knowing all five breast cancer warning signs
- Knowing breast cancer risk factor: Having a close relative with breast cancer
- Knowing breast cancer risk factor: Having type 2 diabetes
- Knowledge of NHS screening programme
- Knowing correct age for first invitation to NHS Breast Screening Programme
- Knowing correct frequency of breast cancer screening invitations

**Cervical cancer knowledge score**

Cervical cancer knowledge score was calculated from the following items, assigning a score of 1 or 0 to each, with the sum giving the total score (out of 5):

- Knowing all four cervical cancer warning signs
- Knowing cervical cancer risk factor: Infection with HPV
- Knowing about NHS vaccination to protect against cervical cancer
- Knowledge of NHS screening programme
- Knowing correct age for first invitation to cervical cancer screening

**Bowel cancer knowledge score**

Bowel cancer knowledge score was calculated from the following items, assigning a score of 1 or 0 to each, with the sum giving the total score (out of 3):

- Knowing all seven bowel cancer warning signs
- Knowing bowel cancer risk factor: Having diabetes
- Knowledge of NHS screening programme

**Total cancer knowledge score**

Total cancer knowledge score was calculated as the sum of Breast, Cervical and Bowel cancer knowledge scores.
