# Supplementary material for: Peer-led, faith-based co-designed intervention to improve Muslim women’s cancer screening: results from the IMCAN study
Source: Front Public Health. 2026 Oct 9;14:1918791. doi: 10.3389/fpubh.2026.1918791 (PMC13690773; doi:10.3389/fpubh.2026.1918791)
Supplement: Supplementary file 2 [file Table_2.DOCX]

**Supplement 2**

List of Barrier belief statements used in analyses:

1. Breast, colorectal or cervical cancer screening is not needed without symptoms
2. I have other more important things to worry about than cancer screening
3. I am not at risk of developing colorectal cancer
4. Colorectal screening is too messy
5. I find it too difficult to complete the colorectal screening test kit
6. I am worried about having to take my clothes off or that too much skin would be showing during cancer screening
7. I am too busy for cancer screening
8. I don't think that I am at risk of cervical cancer
9. I am too embarrassed to go for cancer screening
10. I am too frightened of what a cancer screening test might find
11. I don't want a man to carry out the screening test
12. I am worried about putting extra strain on the NHS / health services
13. The harms of cancer screening outweigh the benefits
14. I cannot afford to cover the costs doing cancer screening (e.g. transport, childcare, reduced pay/earnings)
15. Nothing would put me off a cancer screening test
16. The screening appointment is too far away from my home
