# Supplementary material for: Peer-led, faith-based co-designed intervention to improve Muslim women’s cancer screening: results from the IMCAN study
Source: Front Public Health. 2026 Oct 9;14:1918791. doi: 10.3389/fpubh.2026.1918791 (PMC13690773; doi:10.3389/fpubh.2026.1918791)
Supplement: Supplementary file 3 [file Table_3.DOCX]

**Supplement 3**

## Eligible Participants - Ethnicity

| **Variable** | **Statistic** | **All (n=167^*^)** | **England (n=92)** | **Scotland (n=75)** |
| --- | --- | --- | --- | --- |
| **Ethnicity (n=167)** |  |  |  |  |
| Afghani | N (%) | 3 (1.8%) | 3 (3.3%) | 0 (0.0%) |
| Arab | N (%) | 15 (9.0%) | 14 (15.2%) | 1 (1.3%) |
| Bangladeshi | N (%) | 28 (16.8%) | 28 (30.4%) | 0 (0.0%) |
| Black African | N (%) | 4 (2.4%) | 3 (3.3%) | 1 (1.3%) |
| British Pakistani | N (%) | 3 (1.8%) | 2 (2.2%) | 1 (1.3%) |
| English/Welsh/Scottish/ Northern Irish/British | N (%) | 3 (1.8%) | 0 (0.0%) | 3 (4.0%) |
| Indian | N (%) | 2 (1.2%) | 0 (0.0%) | 2 (2.7%) |
| Iranian | N (%) | 3 (1.8%) | 3 (3.3%) | 0 (0.0%) |
| Pakistani | N (%) | 94 (56.3%) | 32 (34.8%) | 62 (82.7%) |
| Somali | N (%) | 1 (0.6%) | 1 (1.1%) | 0 (0.0%) |
| Sri Lankan | N (%) | 1 (0.6%) | 1 (1.1%) | 0 (0.0%) |
| White and Asian | N (%) | 6 (3.6%) | 3 (3.3%) | 3 (4.0%) |
| White and Black African | N (%) | 1 (0.6%) | 0 (0.0%) | 1 (1.3%) |
| White and Black Caribbean | N (%) | 2 (1.2%) | 1 (1.1%) | 1 (1.3%) |
| Prefer not to say | N (%) | 1 (0.6%) | 1 (1.1%) | 0 (0.0%) |

*Two participants from Scotland did not indicate their ethnicity (missing values)
